# Design of TRUST, a non-interventional, multicenter, 3-year prospective study investigating an integrated patient management approach in patients with relapsing-remitting multiple sclerosis treated with natalizumab

**DOI:** 10.1186/s12883-016-0625-0

**Published:** 2016-07-12

**Authors:** Tjalf Ziemssen, Achim Gass, Jens Wuerfel, Antonios Bayas, Björn Tackenberg, Volker Limmroth, Ralf Linker, Mathias Mäurer, Judith Haas, Martin Stangel, Matthias Meergans, Olof Harlin, Hans-Peter Hartung

**Affiliations:** Department of Neurology, MS Center Dresden, Center of Clinical Neuroscience, University Hospital Carl Gustav Carus, Dresden University of Technology, Fetscherstr. 74, 01307 Dresden, Germany; Department of Neurology, University Medicine Mannheim UMM, University of Heidelberg, Mannheim, Germany; Medical Image Analysis Center (MIAC AG), Basel, Switzerland; Department of Neurology, Hospital Augsburg, Augsburg, Germany; Department of Neurology, Philipps University and University Clinics Gießen and Marburg, Marburg, Germany; Department of Neurology, Cologne General Hospitals, University of Cologne, Cologne, Germany; Department of Neurology, Friedrich-Alexander University Erlangen, Erlangen, Germany; Department of Neurology, Caritas Hospital Bad Mergentheim, Bad Mergentheim, Germany; Department of Neurology, Jewish Hospital, Berlin, Germany; Department of Neurology, Clinical Neuroimmunology and Neurochemistry, Hannover Medical School, Hannover, Germany; Biogen, Zug, Switzerland; Biogen, Ismaning, Germany; Department of Neurology and Center for Neuropsychiatry, Medical Faculty, Heinrich-Heine-University Düsseldorf, Düsseldorf, Germany

**Keywords:** Natalizumab, Relapsing-remitting multiple sclerosis, Progressive multifocal leukoencephalopathy, John Cunningham virus

## Abstract

**Background:**

Natalizumab provides rapid and high-efficacy control of multiple sclerosis disease activity with long-term stabilization. However, the benefits of the drug are countered by a risk of developing progressive multifocal leukoencephalopathy in patients infected with the John Cunningham Virus. Close monitoring is required in patients with increased progressive multifocal leukoencephalopathy risk receiving natalizumab in the long-term for an optimal benefit-risk evaluation. Standardized high-quality monitoring procedures may provide a superior basis for individual benefit and risk evaluation and thus improve treatment decisions. The non-interventional study TRUST was designed to capture natalizumab effectiveness under real-life conditions and to examine alternate approaches for clinical assessments, magnetic resonance imaging monitoring and use of biomarkers for progressive multifocal leukoencephalopathy risk stratification.

**Methods/Design:**

TRUST is a non-interventional, multicenter, prospective cohort study conducted at approximately 200 German neurological centers. The study is intended to enroll 1260 relapsing-remitting multiple sclerosis patients with ongoing natalizumab therapy for at least 12 months. Patients will be followed for a period of 3 years, irrespective of treatment changes after study start. Data on clinical, subclinical and patient-centric outcomes will be documented in order to compare the effectiveness of continuous versus discontinued natalizumab treatment. Furthermore, the type and frequency of clinical, magnetic resonance imaging and biomarker assessments, reasons for continuation or discontinuation of therapy and the safety profile of natalizumab will be collected to explore the impact of a systematic patient management approach and its potential impact on patient outcome. Specifically, the role of biomarkers, the use of expert opinions, the impact of high-frequency magnetic resonance imaging assessment for early progressive multifocal leukoencephalopathy detection and the role of additional radiological and clinical expert advice will be explored.

**Discussion:**

TRUST was initiated in spring 2014 and enrollment is anticipated to be completed by mid 2016. Annual interim analyses will deliver continuous information and transparency with regard to the patient cohorts and the completeness and quality of data as well as closely monitor any safety signals in the natalizumab-treated cohort. The study’s results may provide insights into opportunities to improve the benefit-risk assessment in clinical practice and support treatment decisions.

## Background

Natalizumab (Tysabri^®^) is an intravenous humanized monoclonal antibody directed against α4-integrin (CD49d), a specific adhesion molecule located on the surface of lymphocytes and other immune cells. The binding of natalizumab to its target inhibits the transmigration of lymphocytes across the blood-brain barrier, leading to reduced disease activity in relapsing-remitting multiple sclerosis (RRMS) [1].

**Fig. 1:**
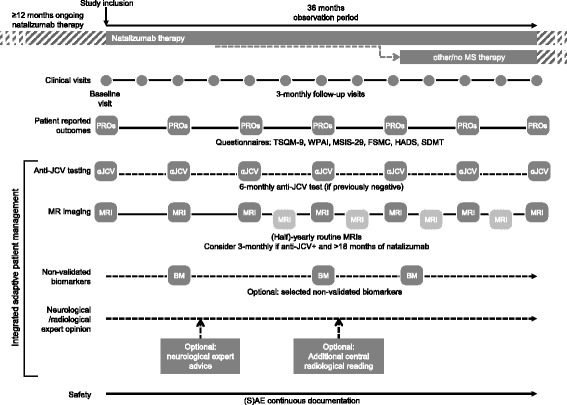
Overview of data documented in TRUST. (S)AE = (serious) adverse event

**Fig. 2:**
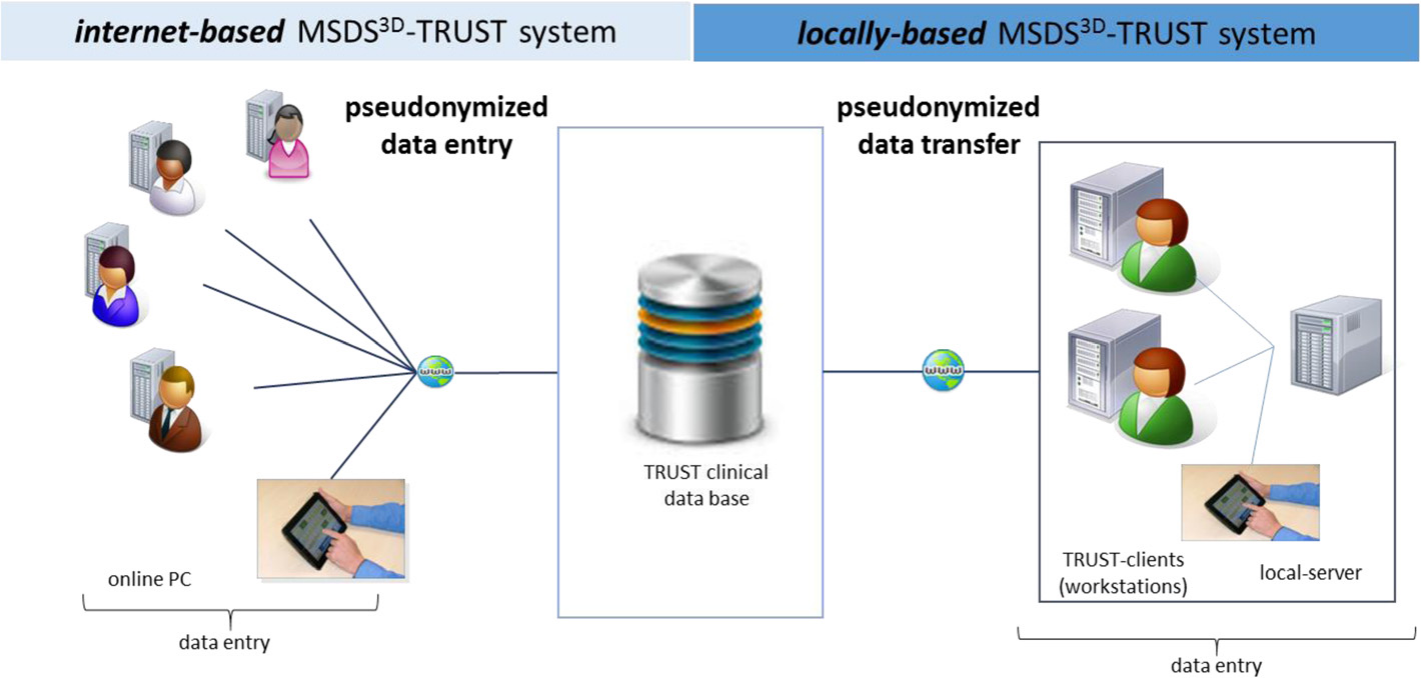
Electronic patient data documentation and management in TRUST. Patient data are captured electronically by the Multiple Sclerosis Documentation System (MSDS^3D^). A specific module of the software has been created for the TRUST study, allowing for electronic entry of pseudonymized visit data and patient questionnaire data. The clinical database and the MSDS^3D^ TRUST software were developed by the MSDS^3D^ Project Group at the Neurological Clinic of the Technical University Dresden, Germany, which is also responsible for data management. Integrated functions in MSDS^3D^-TRUST System include: Visual display of history, events and course of disease, Direct access to requests of neurological expert advice and neuroradiological second opinion, Pseudonymized and individual patient data access for clinical and radiological experts, Straightforward clinical data monitoring for compilation of robust and high-quality results

The efficacy of natalizumab in RRMS was demonstrated in a randomized, double-blind, placebo-controlled 2-year trial. At 2 years, natalizumab reduced the risk of sustained disease progression, as measured by the Expanded Disability Status Scale (EDSS), by 42 %. The annualized relapse rate (ARR) was diminished by 68 %, and the number of new or newly enlarging T2-weighted (T2w) lesions and the number of gadolinium-enhancing lesions on T1-weighted MRI were significantly lower compared to placebo (83 and 92 %, respectively) [2].

Natalizumab was reintroduced in 2006 by the Food and Drug Administration (FDA) and first licensed by the European Medicines Agency (EMA) in 2006, substantially improving treatment options in RRMS patients with poorly controlled disease activity [3]. Long-term data, including clinical observations for up to 5 years of treatment with natalizumab, confirm its sustained effect on disease activity [4]. A retrospective matched-paired analysis strongly suggested natalizumab’s superior efficacy over fingolimod [5]. The greatest observed benefit of natalizumab has been as first-line MS therapy in patients with EDSS values <3 and lower relapse rates at the start of therapy.

To date, more than 149,000 patients have been treated with natalizumab. Natalizumab’s generally favorable safety profile is based on more than 475,000 patient-years of drug exposure [6, 7]. However, the risk of developing progressive multifocal leukoencephalopathy (PML), an often severely disabling disease, limits the use of natalizumab, and a careful benefit-risk assessment for each patient must be continuously performed by the treating physician. PML had been reported most prominently in HIV patients and emerged in MS patients after natalizumab was first introduced in 2004 [8–10]. Sporadic cases of PML have also been reported in MS patients receiving fingolimod or dimethyl fumarate irrespective of pretreatment with natalizumab [11, 12].

The causative agent of PML is the John Cunningham virus (JCV). The risk of developing PML is significantly increased in natalizumab-treated patients who (i) are anti-JCV antibody seropositive, (ii) have received natalizumab treatment for 2 or more years and (iii) have been exposed previously to immunosuppressants [13]. Recently, the correlation of PML-Risk and the JCV-Index has been proven as an additional risk factor in patients without previous immunosuppressive treatment (14), which has led to new recommendations of the European medical regulatory authority [14] Some anti-JCV–seronegative patients demonstrate fluctuating antibody status over time, and the risk of PML in these patients is unclear, although most seroconverters appear to have low levels of anti-JCV antibodies [15].

Recent studies on immunological biomarkers indicate that some of them could be used to further stratify the risk of PML in patients treated with natalizumab [16]. The JCV antibody index is determined by comparing the levels of antibodies of a specific patient to a reference cohort. Analyses from anti-JCV antibodies positive patients with no prior IS treatment suggests that lower index values are associated with a lower risk of developing PML [17].

Measuring L-selectin expression on cryopreserved CD4+ T cells as well as soluble L-selectin in plasma has gained attention lately [17, 18]. However, its usage in clinical practice is hampered by difficulties in sample handling, leading to problems in obtaining consistent results, and is not reliable for assessing PML risk [18–20]. Initial data suggest that the presence of oligoclonal lipid-specific IgM in CSF may be associated with a lower PML risk [21]. In addition, research demonstrating the down regulation of CD49 and CD29 on CD4+ and CD8+ T cells, as well as CD11a and CXCR3, reveals the impact of natalizumab treatment on a variety of cell membrane markers. However, all these novel biomarkers have yet to be validated in the clinical setting [18].

Currently, the diagnosis of PML is based on clinical symptoms, typical PML lesions detected by MRI and the presence of JCV DNA in cerebrospinal fluid and/or biopsy of brain tissue [22]. Published studies indicate that the detection of PML at an early, asymptomatic stage is associated with a better prognosis [23]. Therefore, high-sensitivity quantitative polymerase chain reaction testing should be performed by qualified laboratories if PML is suspected [22]. In addition, MRI protocols such as high-frequency MR monitoring with adjusted sequences and image evaluation by experienced radiologists have been suggested to facilitate the detection of PML (Table 1) [24].

**Table 1:** MAGNIMS consensus guideline: regimens for risk-based MRI monitoring in MS patients treated with natalizumab [24]

Population	Recommended MRI regimen	Interval
Natalizumab-treated patients at high risk of PML (ie, anti-JCV seropositive AND treatment duration ≥18 months)	• Conventional T2-weighted imaging • T2-FLAIR • DWI • Contrast-enhanced T1-weighted imaging [in patients with suspected PML lesions]	• Every 3–4 months
Natalizumab-treated patients at low risk of PML (i.e., anti-JCV seronegative)	• Conventional T2-weighted imaging • T2-FLAIR • DWI	• Every 12 months
Patients who switch from natalizumab to other drugs (including fingolimod, alemtuzumab, dimethyl fumarate)	• Conventional T2-weighted imaging • Contrast-enhanced T1-weighted imaging	• Every 3–4 months, for up to 12 months
Patients at high risk of developing opportunistic infections who are switching immunotherapies	• Conventional T2-weighted Imaging • Contrast-enhanced T1-weighted imaging • T2-FLAIR • DWI • Contrast-enhanced T1-weighted imaging [in patients with suspected PML lesions]	• At end of ongoing therapy • After starting therapy with the next drug

*DWI* diffusion-weighted imaging, *FLAIR* fluid-attenuated inversion recovery

Real-life patient management, PML risk stratification using biomarkers and early detection of PML via MRI monitoring and clinical screening are handled in a variety of ways. Furthermore, natalizumab treatment is often discontinued due to reasons other than breakthrough disease, and a return of disease activity to baseline levels is observed in most patients [25–27]. Long-term, well-structured patient monitoring is thus required to enable individualized treatment approaches and decisions [28]. McGuigan et al. recently proposed consensus expert guidance for in-practice risk management [29], and tools simplifying the implementation of such approaches, such as the multidimensional Multiple Sclerosis Documentation System (MSDS^3D^), are increasingly being used to monitor MS patients [30]. More widespread use of the available instruments may benefit patients treated with natalizumab. However, little is known about the utility of risk-adapted diagnostics and their use for monitoring and treatment decision making in the routine clinical setting.

Management of MS patients in Germany is highly heterogeneous. Treating physicians have diverse backgrounds–varying from academians at institutions with international reputations in the field of MS to office-based neurologists with a minor focus on MS. Many neurologists treating MS patients are organized in regional networks. This allows them to obtain advice (e.g., from academic institutions) for individual patient care. Therefore, the use of expert opinions as an additional element to aid in decision making, with respect to management of natalizumab-treated patients, may be another significant aspect of routine care.

In routine settings, natalizumab is frequently discontinued in patients with two of the established risk factors (anti-JCV antibody positive and at least 2 years of natalizumab therapy) without performing an individualized benefit-risk assessment, thus potentially exposing these patients to a heightened risk of MS relapses and accelerated disease progression [27, 31]. To date, the consequences of a treatment change following natalizumab discontinuation have been examined only in smaller studies, with a limited number of centers involved and follow-up duration. There is, therefore, a need to better understand the reasons for treatment change and its merits and disadvantages under real-life circumstances. Furthermore, JCV-positive patients switching from natalizumab to dimethyl fumarate or fingolimod are not totally safe in regard to contracting PML.

The non-interventional study TRUST (TysabRi^®^ patient management via a longitUdinal multidimensional STudy) was designed primarily to capture the real-life efficacy of natalizumab in patients on long-term therapy but also to understand the range of approaches to PML risk stratification via clinical assessments, MRI monitoring and use of candidate biomarkers and expert advice, and the utility of adaptive integrated management tools. The results may provide insights to improve the benefit-risk assessment in clinical practice and support treatment decisions.

In this article we report the design, methods and procedures of this study.

## Methods/Design

### Study goals and scope

The major study goals of TRUST are (i) to assess the course of disease over 36 months in patients with sustained natalizumab therapy versus those who discontinue natalizumab treatment; (ii) to evaluate the use and effects of an integrated adaptive patient management approach, including multimodal monitoring and utilization of expert advice; (iii) to assess the factors affecting the decision to continue or discontinue natalizumab treatment; (iv) to explore patient-centric outcomes in patients continuing versus discontinuing natalizumab treatment.

TRUST includes patients with RRMS treated with natalizumab for at least 12 months, regardless of their duration of disease. The study will collect information that is routinely acquired in clinical practice in Germany. Patients will be followed for 3 years, irrespective of whether natalizumab treatment is continued or discontinued, providing information to assess the efficacy of natalizumab in a real-life setting (Fig. 1). Furthermore, factors influencing the decision to continue or discontinue natalizumab will be documented. Lastly, TRUST will evaluate the utility of integrated adaptive patient management approaches for monitoring patients with RRMS with respect to disease activity, discontinuation of therapy and patient safety.

The non-interventional observational prospective study design allows the exploration of the use of PML risk-related biomarkers such anti-JCV-Antibody status and - Index in Serum [15] as well as the utility of newer, non-validated biomarkers, such as JCV-Antibody index in serum/CSF [32] and lipid specific IgM bands [21] in treatment decisions and of MRI for early detection of PML in a real-world setting. Furthermore, patient-centric outcomes will be captured in order to understand the impact of different treatment alternatives on the patients’ daily situation.

### Study population and sample size

TRUST will include a total of 1260 patients from approximately 200 centers in Germany who fulfill the following criteria: at least 18 years of age at inclusion; diagnosis of RRMS according to McDonald criteria (2010 version) [33]; treatment with natalizumab according to prescription information for at least 12 months and informed consent prior to participating in the study.

Patients with any of the following characteristics will be excluded from the study: progressive forms of MS; participation in the non-interventional Tysabri Observational Program (TOP) [3] (patients included in the German REGIMS registry [34] may participate); simultaneous therapy with another drug indicated for the treatment of RRMS or contraindications to natalizumab treatment according to prescription information.

Patient recruitment in TRUST was initiated in August 2014. The planned duration for recruitment is 22 months. Currently, approximately 170 neurologists, 120 radiologists and 1000 patients are participating in the study. The geographic distribution of centers throughout Germany follows the population density. About two-thirds of the centers are office-based neurologists and one-third are hospitals, including eight large academic institutions. The planned duration of observation period per patient is 36 months. Data acquisition is expected to be completed in 2019.

Due to the study design, a high level of drop out is expected. Hence, assuming a Poisson distribution and 3 years of follow-up,1260 patients will be included in the study to adjust for study dropouts and patients restarting natalizumab treatment in order to detect differences with an alpha level of 0.05.

### Procedures

Data are acquired using the MS patient management and documentation software package MSDS^3D^. A specific module of the MSDS^3D^ software platform was developed for TRUST that can be used either as a local application or as an online web interface (Fig. 2). The MSDS^3D^ is currently used for the documentation in the REGIMS registry of the Competence Network Multiple Sclerosis (KKNMS) and the German MS Society (DMSG) Registry.

Patient data are documented using electronic reporting forms. Patient-centric outcomes are entered in questionnaires.

Neurologists who make use of clinical expert advice at any time during the study and radiologists seeking an MRI second opinion reading are asked to document such processes.

Study procedures are intended explicitly to not interfere with or exert influence on therapeutic decisions made by the treating neurologist.

### Data acquisition

Data and measurements recorded in TRUST are listed in Table 2. Patient data are recorded at study entry (baseline/visit #1) and at 12 subsequent visits (visit #2 through #13, conducted approximately every 3 months until month 36 after baseline). Visits and measurements are performed within the framework of routine clinical care without causing any additional burden to the patient.

**Table 2:** Data acquisition plan for TRUST

Documented items	Baseline (visit #1)	Approximately every 3 months (visits #2–13) until month 36
Definition of investigator’s infrastructure	x	
Informed consent	x	
Patient demographic characteristics	x	
History (medical, MS):	x	
First MS diagnosis		
Other underlying diseases		
History of EDSS score		
History of relapses		
History of (previous) MS therapy		
MRI status at baseline		
Opportunistic infections before start of natalizumab treatment		
History of natalizumab treatment	x	
Status of current treatment		x
Current EDSS score	x	x
Relapse(s) since last visit (number, date)		x
AE at/since (last) visit (yes, no)	x	continuously
Anti-JCV serostatus		x
Other biomarkers collected or evaluated (e.g. anti-JCV antibody index)		x
Resources used (nurse contact, other HCP, clinic)		x
Lymphocytes and other critical laboratory parameters in association with natalizumab treatment	x	x
Opportunistic infections		x
TSQM-9 (treatment satisfaction)	x	every 12 months
FSMC (fatigue)	x	every 12 months
MSIS-29 (MS-related quality of life)	x	every 12 months
HADS (depression)	x	every 6 months
SDMT (cognitive function)	x	every 6 months
WPAI (productivity)	x	every 6 months
Expert advice requested		if available
MRI results	x	if available

Due to the non-interventional character of the study, only data collected within the routine clinical practice are documented

*TSQM-9* Treatment Satisfaction Questionnaire for Medication, *FSMC* Fatigue Scale for Motor and Cognitive Functions, *MSIS-29* Multiple Sclerosis Impact Scale-29 items, *HADS* Hospital Anxiety and Depression Scale, *SDMT* Symbol Digit Modality Test, *WPAI* Work Productivity and Activity Impairment Questionnaire

Patient-related data are exclusively recorded and processed in pseudonymized formats. The clinical database is managed by the Center of Clinical Neuroscience, University of Dresden, the imaging database in a joint project by mediri GmbH and the University Goettingen (JW). Electronic data transfer to the TRUST study server is SSL encrypted. National data privacy laws apply to all data processing.

Ongoing documentation independent of visit dates includes changes/interruptions in natalizumab or other MS therapy, changes in co-medications, reporting of adverse events (AEs) and pregnancies and utilization of expert advice.

#### Patient-centric outcomes

Patient-centric outcomes are captured through the following six instruments: treatment satisfaction (Treatment Satisfaction Questionnaire for Medication [TSQM-9]), fatigue (Fatigue Scale for Motor and Cognitive Functions [FSMC]), MS-related quality of life (Multiple Sclerosis Impact Scale-29 items [MSIS-29]), depression (Hospital Anxiety and Depression Scale [HADS]), cognitive function (Symbol Digit Modality Test [SDMT]) and work productivity/activity (Work Productivity and Activity Impairment Questionnaire [WPAI]). Questionnaires are administered at baseline and every 6 months during follow-up. Patients fill in the questionnaires using tablet computers or in paper-and-pencil format with the exception of the SDMT, which is administered by the site staff as a paper-based test. All data are transferred into the TRUST database either directly from the tablet or after digitalization from paper-based entries.

#### Documentation at end of follow-up

At the end of follow-up, i.e. at the end of study participation, reasons for premature study discontinuation (if applicable), discontinuation or continuation of natalizumab, type of next therapy and patient questionnaire entries are recorded.

#### Adverse events

An AE is defined as any unfavorable change in the patient’s pretreatment condition, regardless of a potential relation to treatment and irrespective of whether the medication was used as prescribed. AEs are reported independently from the planned study visits using an AE report form in the TRUST module of MSDS^3D^-TRUST. Serious adverse events (SAEs) include lethal or life-threatening events, hospitalizations, events leading to major incapacity, persistent or significant disability, congenital anomaly or birth defects and events that are otherwise medically significant. Abnormal laboratory values and test results may also be considered SAEs. SAEs must be reported within 24 h after their recognition to the manufacturer of natalizumab. In cases where indications of suspected PML is revealed within the frame of the optional clinical or imaging expert advice process, treating neurologists and (neuro-)radiologists are notified within 24 h.

#### Documentation of expert advice utilization

##### Clinical expert advice

If a clinical opinion from an expert neurologist or radiologist is requested, the requesting physician is asked to document his or her satisfaction with the timing and content of the advice.

##### Imaging expert advice

Neuroradiological centers may optionally request an MRI expert advice second opinion as part of the clinical routine. For this purpose, anonymized patient data is transferred per DICOM (Digital Imaging and Communications in Medicine) to a database separate from the clinical database. Patient identification and data quality checks are performed prior to image reading according to a prespecified protocol. The MRI database and MRI quality assurance processes used in TRUST have been developed by the University of Göttingen in cooperation with the Medical Imaging Research Institute (mediri), which is responsible for MRI data management and the optional MRI second opinion reading process.

### Quality control

Quality control in TRUST aims at ensuring a complete dataset as detailed in the recommendations for planning, conduct and analysis of non-interventional observational studies issued by the German Federal Institute for Drugs and Medical Devices [35]. Plausibility and completeness of data are checked during entry. Missing information and discrepancies are clarified via queries to the individual study center according to a predefined data validation plan. The conduct of the study is supervised by monitors of the contract research organization according to a predefined monitoring plan. The data management center examines the data transferred from the centers for potential unreported adverse events.

### Reporting and data analyses

On completion of documentation, all recorded data will be analyzed using descriptive statistics according to the recommendations for observational studies [36]. Yearly interim analyses are planned. Data capturing and corresponding quality is monitored in the course of the observational trial.

ARR for patients treated with natalizumab during the complete follow-up period (36 months) and those with earlier discontinuation of natalizumab and subsequent alternative MS treatment will be calculated. All endpoints for patient-centric outcomes will be analyzed according to the usual score calculations. Subgroup analyses according to demographic variables, EDSS scores, relapse rates, biomarker status, MRI findings and natalizumab treatment duration will be performed. AEs and data on safety-related biomarkers captured in the daily practice routine will be reported descriptively. Further explorative analysis and subgroups will be defined in a statistical analysis plan prior the database lock. Correlations of disease or treatment characteristics to clinical parameters will be investigated

### Ethical aspects

The study advisory committee advises on study design and data analysis. An independent data monitoring committee is responsible for review of the ongoing safety of patients enrolled in the study. TRUST has been approved by the responsible ethics committees at Heinrich Heine University Düsseldorf (study number: 4675). Regional ethics committees are consulted in accordance with both the codex of the Voluntary Self-Regulation of the Pharmaceutical Industry (FSA) [37] and recommendations dealing with quality aspects of non-interventional observational studies issued by the German Federal Institute for Drugs and Medical Devices (BfArM) [35]. Patients may withdraw their consent to study participation at any time without detrimental consequences for their further treatment and care.

### Publication

The results of the TRUST study will be published in agreement with the study advisory committee and according to the rules of the International Committee of Medical Journal Editors and Good Publication Practice [38].

## Discussion

Here we report on the study design of the prospective, multicenter, non-interventional, long-term cohort study TRUST. The study should improve the understanding of the use and discontinuation of natalizumab in the routine clinical practice setting, the influence of natalizumab on MS disease course, the effectiveness of sustained natalizumab treatment and the utility of risk-management and surveillance tools as well as expert opinions in the real-life setting.

Currently, observations on the return of MS disease activity after discontinuation of natalizumab have been described in one prospective randomized clinical trial by Fox et al [39]. Patients with stable disease status on natalizumab therapy were randomized to continue natalizumab treatment or switch to interferon beta, glatiramer acetate or corticosteroid treatment. The results indicated increased MS disease activity after switching to alternative treatments [39]. Further investigations in similar settings, with treatment switched to fingolimod or dimethyl fumarate, showed equal trends [26, 40]. To date, the follow-up periods of these investigations have been limited and information on the impact on disability progression is lacking. The TRUST study allows for longer follow-up and investigation under real-life conditions. Moreover, the utilization and the diagnostic and predictive value of risk stratification approaches (anti-JCV antibody serostatus and index as well as additional non-validated biomarkers), as well as the use of MRI for the early detection of PML, have not been assessed to date in patients on long-term natalizumab treatment or in those switching to other MS therapies in routine clinical practice.

Due to the chronic course of MS disease with accumulating disability and the need for long-term therapy, patients with MS generally require close medical attendance. Close monitoring is required for patients receiving natalizumab to optimize their individual benefit-risk ratio [41]. Evaluating the impact of the JCV antibody index on treatment decisions in this patient cohort in TRUST will be informative with respect to benefit-risk considerations. Furthermore, based on current PML risk estimates some of the patients enrolled in TRUST may serve as an important control group for an assessment of disease activity after 2 years of therapy.

Interdisciplinary collaboration as well as expert consultation are options commonly used in MS patient management. This study therefore explores the use of expert advice from experienced MS physicians to evaluate its impact on treatment decisions.

The results of this study are expected to contribute to the improvement of MS patient management and to facilitate rational treatment decisions in patients receiving long-term therapy with natalizumab.

## Abbreviations

AE, adverse event; ARR, annualized relapse rate; BfArM, German Federal Institute for Drugs and Medical Devices; DMSG, German MS Society; EDDS, Expanded Disability Status Scale; EMA, European Medicines Agency; FDA, Food and Drug Administration; FSA, Voluntary Self-Regulation of the Pharmaceutical Industry; FSMC, Fatigue Scale for Motor and Cognitive Functions; HADS, Hospital Anxiety and Depression Scale; IS, immunosuppressive; JCV, John Cunningham virus; KKNMS, Competence Network Multiple Sclerosis; MSDS, Multiple Sclerosis Documentation System; MSIS-29, Multiple Sclerosis Impact Scale-29 items; PML, progressive multifocal leukoencephalopathy; RRMS, relapsing-remitting multiple sclerosis; SAE, serious adverse event; SDMT, Symbol Digit Modality Test; T2w, T2-weighted; TSQM-9, Treatment Satisfaction Questionnaire for Medication; WPAI, Work Productivity and Activity Impairment Questionnaire
